# Macrolide resistance trajectories across three *Bordetella pertussis* genetic backgrounds under stepwise erythromycin exposure

**DOI:** 10.3389/fmicb.2026.1803864

**Published:** 2026-04-17

**Authors:** Kaichong Jiang, Wei Wang, Wenjuan Zhao, Yang Luan, Da Xue, Shuyue Tang, Xiao Ma, Zengguo Wang

**Affiliations:** 1 National Regional Children’s Medical Center (Northwest), Shaanxi Institute for Pediatric Diseases, Xi’an Children’s Hospital, Affiliated Children’s Hospital of Xi’an Jiaotong University, Xi'an, Shaanxi, China; 2 Department of Clinical Laboratory, National Regional Children’s Medical Center (Northwest), Xi’an Children’s Hospital, Affiliated Children’s Hospital of Xi’an Jiaotong University, Xi'an, Shaanxi, China; 3 School of Public Health, Shaanxi University of Chinese Medicine, Xianyang, Shaanxi, China; 4 Xi’an Center for Disease Control and Prevention, Xi’an, Shaanxi, China; 5 Department of Diphtheria, Tetanus and Pertussis Vaccine and Toxins, National Institute for Food and Drug Control, Beijing, China

**Keywords:** *Bordetella pertussis*, collateral sensitivity, genetic screen, low-heterogeneity bacterial model, resistance

## Abstract

**Background:**

Macrolide resistance in *Bordetella pertussis* remains a clinical concern, yet how broader genomic background may be associated with resistance-associated trajectories under antibiotic exposure is not fully understood.

**Methods:**

We performed stepwise erythromycin (ERY) induction using three clinical macrolide-susceptible parental isolates representing clinically relevant genetic backgrounds. Resistance-associated variants were tracked by whole-genome sequencing together with phenotypic readouts, including antimicrobial susceptibility testing, growth and biofilm assays, and ELISA-based measurements of selected virulence- and metabolism-associated factors.

**Results:**

ERY minimum inhibitory concentration (MIC) trajectories diverged across genetic backgrounds. B19068 and B181 reached high-level resistance and exhibited a 23S rRNA G2046A substitution in the consensus sequence, whereas B197 followed a lower-MIC trajectory and accumulated variants in efflux-associated loci. Whole-genome sequencing further revealed genomic alterations across genetic backgrounds, including structural variation in a subset of isolates, indicating that serial passaging under antibiotic exposure can be accompanied by genomic alterations beyond candidate resistance loci. Antibiotic-exposed lineages also displayed differences in growth and biofilm formation across genetic backgrounds, and ELISA readouts (PT, FHA, LPS, DHFS, and DHFR) differed across backgrounds under the *in vitro* conditions used.

**Conclusion:**

Under a controlled induction regimen, three representative clinical genomic backgrounds exhibited divergent macrolide-resistance trajectories and accompanying phenotypic differences. These observations are consistent with background-linked differences in resistance evolution and provide a basis for future validation in replicated evolution experiments.

## 1 Introduction

Antibiotic resistance poses a growing challenge to effective antimicrobial therapy, driven by the accumulation and spread of resistance-associated genetic variants in bacterial populations. These resistance genes may be located on chromosomes, plasmids, or ribosomes, and even appear in combination within the strain as multidrug-resistant bacteria (Lange et al., 2019). Moreover, the sources of resistance vary between the environment with low concentrations of antibiotics and in populations where heterogeneously resistant strains emerge, followed by antibiotic selection (Oz et al., 2014). These issues substantially affect the control of antibiotic resistance. Directly observing evolution in natural settings is challenging and time-consuming (Behe, 2010). Therefore, to study bacterial resistance, researchers often use genetic screens to identify resistance mutations, which provide critical data on how bacteria develop antibiotic resistance and undergo phenotypic changes (Rainey et al., 2017). Many researchers have explored the molecular mechanisms of antibiotic resistance through genetic screening, revealing how bacteria develop resistance through various mutations (Knopp and Andersson, 2018; Cooper, 2018). Previous studies have demonstrated that bacteria can accumulate mutations that contribute to antibiotic resistance over time.

Many culturable bacteria have been used to study antibiotic-resistance evolution. However, most research focuses on fast-growing bacteria with manageable genomes, while others use strains that can be genetically engineered to either delete (McCloskey et al., 2018) or modify genes (Kacar et al., 2017). These studies typically explore aspects such as gene regulation, antibiotic resistance, and host-microbiome interactions (Rainey et al., 2017; Long et al., 2015; Martin et al., 2016; Bruger and Marx, 2018). In contrast, fewer studies focus on slow-growing bacteria, such as *Bordetella pertussis*, that are more challenging to genetically modify but offer distinct advantages for studying resistance mechanisms.

*Bordetella pertussis* is often described as having relatively limited within-lineage diversity, which can facilitate identification of resistance-associated variants under defined conditions (Xu et al., 2015). However, clinically prevalent genotypes (e.g., *ptxP1*- and *ptxP3*-associated backgrounds) can differ at numerous loci, and such differences have the potential to contribute to variation in phenotypic readouts, potentially involving epistatic interactions. Its highly conserved genome and low genetic heterogeneity make it a useful model for studying the genetic basis of antibiotic resistance under defined experimental conditions, as it allows for precise tracking of genetic mutations. Previous work has shown that macrolide-resistance associated genetic changes can be recovered in several *Bordetella* spp. under antibiotic exposure, including *B. holmesii* and *B. parapertussis* (Fong et al., 2022). Differences in genome size and gene content between these species could alter the available mutational targets and regulatory context for resistance. Motivated by these observations, we performed a stepwise erythromycin induction experiment in clinically relevant *B. pertussis* backgrounds to explore resistance-associated variants and accompanying phenotypes under a controlled regimen.

In both Western countries and China, *B. pertussis* populations show distinct patterns in their genetic backgrounds of major genomic backgrounds. While Western countries predominantly have *ptxP3*/*fhaB1*-macrolide-susceptible *B. pertussis* (MSBP) (Bart et al., 2014), macrolide-resistant *B. pertussis* in China has traditionally been associated with *ptxP1*/*fhaB3* backgrounds, although recent surveillance suggests a growing presence of *ptxP3*/*fhaB1* lineages (Yao et al., 2020; Cai et al., 2023). Macrolide resistance in *B. pertussis* is mainly based on the 23S rRNA A2047G mutation in clinical MRBP strains, with a few other reported resistance mechanisms (Li et al., 2025). This raises the question of whether different genetic backgrounds influence how resistance develops. In this study, we selected three clinical macrolide-susceptible parental isolates carrying clinically relevant *ptxP*/*fhaB* allele combinations to represent distinct genomic backgrounds for erythromycin induction. These parental isolates served as comparative backgrounds for examining resistance-associated genetic and phenotypic changes during erythromycin induction.

## 2 Materials and methods

### 2.1 Bacterial strains and growth conditions

The strains used in this study included one *B. pertussis* ATCC reference strain and eight clinical isolates (Table 1): three *ptxP3*/*fhaB1*-MRBP isolates and three *ptxP1*/*fhaB3*-MRBP isolates (including previously characterized strains and three isolates collected in 2024), together with two macrolide-susceptible parental strains selected to match these major *ptxP*/*fhaB* backgrounds (B19068M0 and B181M0) and an additional *ptxP1*/*fhaB1*-MSBP isolate (B197M0). The clinical MRBPs were selected to share the same *ptxP*/*fhaB* allele combinations as the parental strains used for induction, in order to provide clinically relevant comparators within each lineage background. Clinical isolates were analyzed in parallel as reference comparators to contextualize the phenotypic and antimicrobial susceptibility profiles observed in the induction-derived lineages, rather than serving as part of the induction experiment. For all serial passaging, charcoal agar (OXOID, CM0119) plates were used. To reduce unintended laboratory adaptation outside the intended induction procedure, isolates were routinely revived from −80 °C stocks and handled with minimal propagation prior to downstream testing, avoiding unnecessary serial subculture. For assays requiring liquid culture, Stainer and Scholte (SS) broth was used (with FeSO_4_·7H_2_O, L-cysteine, L-ascorbic acid, nicotinic acid, reduced L-glutathione, and cyclodextrin). Unless otherwise mentioned, all cultures were performed at 37 °C; liquid cultures in SS broth were incubated with shaking at 230 rpm. Plates were inspected daily; incubation times were extended as needed to ensure visible colony formation from low-inoculum or stressed samples.

**Table 1:** The genotype and erythromycin minimum inhibitory concentration (MIC) of strains.

Species	Strains	Strain source	Genotype	Erythromycin MIC
*Bordetella pertussis*	ATCC9797	Standard strain	*ptxA1/fhaB1/ptxP1*	<0.016
*Bordetella pertussis*	B197M0	Clinical isolate	*ptxA1/fhaB1/ptxP1*	<0.016
*Bordetella pertussis*	B197M0.25	Antibiotic-exposed strain	*ptxA1/fhaB1/ptxP1*	0.25
*Bordetella pertussis*	B197M0.5	Antibiotic-exposed strain	*ptxA1/fhaB1/ptxP1*	0.5
*Bordetella pertussis*	B197M1	Antibiotic-exposed strain	*ptxA1/fhaB1/ptxP1*	1
*Bordetella pertussis*	B197M4	Antibiotic-exposed strain	*ptxA1/fhaB1/ptxP1*	4
*Bordetella pertussis*	B197M12	Antibiotic-exposed strain	*ptxA1/fhaB1/ptxP1*	12
*Bordetella pertussis*	B197M32	Antibiotic-exposed strain	*ptxA1/fhaB1/ptxP1*	32
*Bordetella pertussis*	B197MC5	B197M32 Cultivation for more than 5 generations with no resistance	*ptxA1/fhaB1/ptxP1*	32
*Bordetella pertussis*	B19068M0	Clinical isolate	*ptxA1/fhaB1/ptxP3*	<0.016
*Bordetella pertussis*	B19068M0.25	Antibiotic-exposed strain	*ptxA1/fhaB1/ptxP3*	0.25
*Bordetella pertussis*	B19068M0.5	Antibiotic-exposed strain	*ptxA1/fhaB1/ptxP3*	0.5
*Bordetella pertussis*	B19068M1	Antibiotic-exposed strain	*ptxA1/fhaB1/ptxP3*	1
*Bordetella pertussis*	B19068M24	Antibiotic-exposed strain	*ptxA1/fhaB1/ptxP3*	24
*Bordetella pertussis*	B19068M256	Antibiotic-exposed strain	*ptxA1/fhaB1/ptxP3*	>256
*Bordetella pertussis*	B19068MC5	B19068M256 Cultivation for more than 5 generations with no resistance	*ptxA1/fhaB1/ptxP3*	>256
*Bordetella pertussis*	B181M0	Clinical isolate	*ptxA1/fhaB3/ptxP1*	<0.016
*Bordetella pertussis*	B181M0.25	Antibiotic-exposed strain	*ptxA1/fhaB3/ptxP1*	0.25
*Bordetella pertussis*	B181M0.5	Antibiotic-exposed strain	*ptxA1/fhaB3/ptxP1*	0.5
*Bordetella pertussis*	B181M1	Antibiotic-exposed strain	*ptxA1/fhaB3/ptxP1*	1
*Bordetella pertussis*	B181M256G1	Antibiotic-exposed strain	*ptxA1/fhaB3/ptxP1*	>256
*Bordetella pertussis*	B181M256G2	Antibiotic-exposed strain	*ptxA1/fhaB3/ptxP1*	>256
*Bordetella pertussis*	B181MC5	B181M256G2 Cultivation for more than 5 generations with no resistance	*ptxA1/fhaB3/ptxP1*	>256
*Bordetella pertussis*	BP240705115	Clinical isolate	*ptxA1/fhaB1/ptxP3*	>256
*Bordetella pertussis*	BP240705113	Clinical isolate	*ptxA1/fhaB1/ptxP3*	>256
*Bordetella pertussis*	BP240704125	Clinical isolate	*ptxA1/fhaB1/ptxP3*	>256
*Bordetella pertussis*	BP19225	Clinical isolate	*ptxA1/fhaB3/ptxP1*	>256
*Bordetella pertussis*	BP19234	Clinical isolate	*ptxA1/fhaB3/ptxP1*	>256
*Bordetella pertussis*	BP19147	Clinical isolate	*ptxA1/fhaB3/ptxP1*	>256

*MIC, Minimum inhibitory concentration.

### 2.2 Selection and isolation of erythromycin-resistant mutants

**Figure 1:**
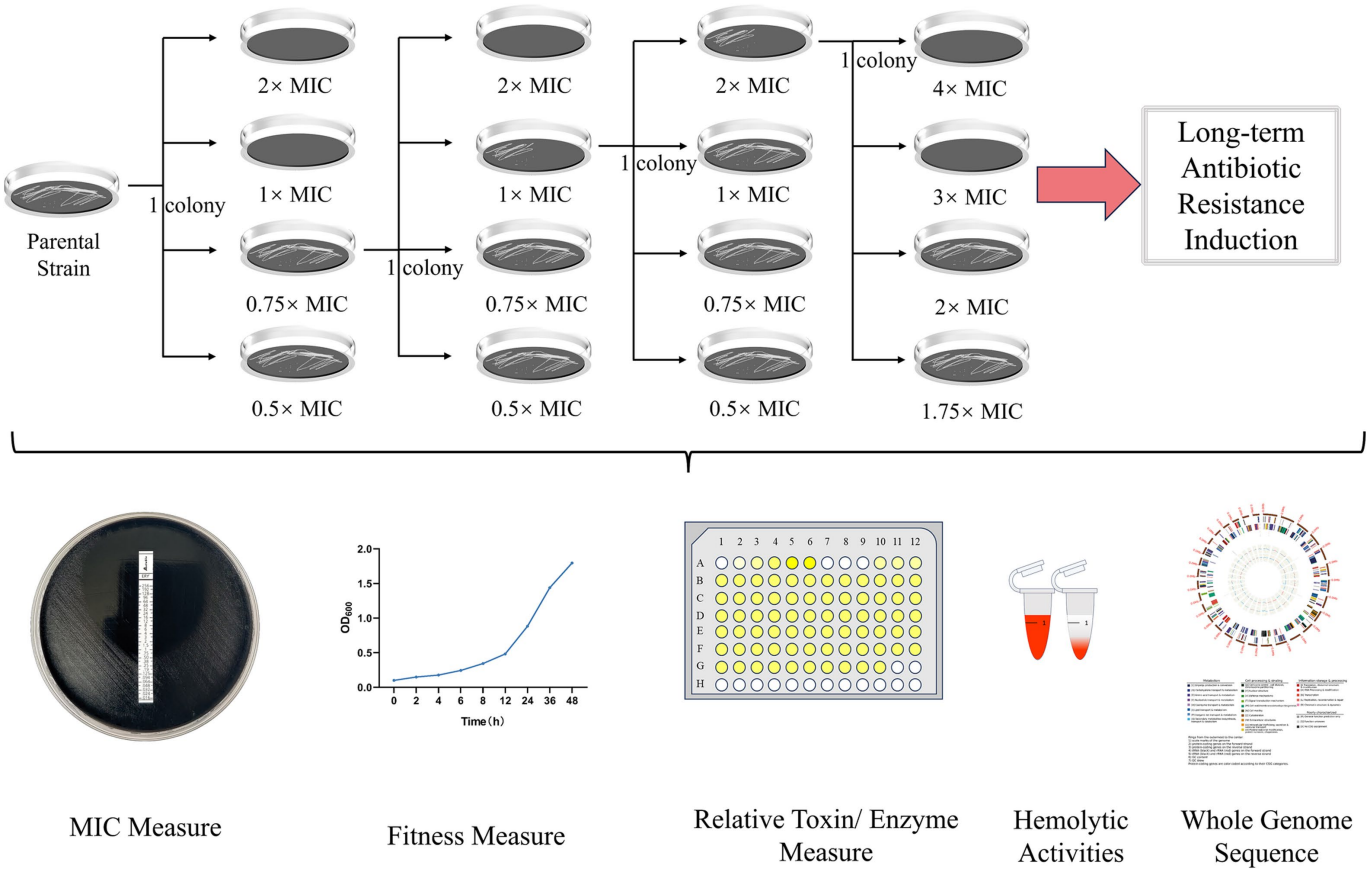
Overview of the induction workflow and downstream assays. Clonal lineages were serially passaged under stepwise erythromycin selection at 0.5×, 0.75×, 1×, and 2× the current MIC. Representative isolates from key induction stages were subjected to whole-genome sequencing/assembly and phenotypic profiling, including erythromycin MIC determination, toxin readouts, and additional susceptibility/fitness-related assays. MIC, minimum inhibitory concentration.

A schematic overview of the stepwise induction procedure is shown in Figure 1. We subjected single-colony–derived isolates of three macrolide-susceptible parental strains (B197M0, B19068M0, and B181M0) to serial passaging under stepwise erythromycin (ERY) exposure to generate antibiotic-exposed lineages for downstream analysis. For each parental strain, induction was carried out as a single passaging series rather than in multiple parallel lineages The strains were revived from −80 °C stocks and passaged for two generations in batch culture to standardize the inoculum prior to ERY exposure. After this standardization, each isolate was serially passaged with ERY increased stepwise from 0.5 × MIC to 2 × MIC, and isolates from defined ERY steps were collected and archived for downstream analyses. At each passage, approximately 10^4^ CFU were transferred to maintain a consistent inoculum size across steps. The ERY concentration was adjusted based on the observed phenotype of the induced isolates at each step. Continuous passaging was conducted until resistance-associated variants were detected and the target MIC levels were achieved. At each step, an aliquot was archived at −80 °C to preserve the strain at that stage and to minimize additional passage-associated changes during downstream assays. After candidate variants were identified, the final antibiotic-exposed (AE) strains were passaged on antibiotic-free charcoal agar for more than five generations to assess phenotypic stability. The strains were then preserved for further experiments. This induction approach is distinct from population-based experimental evolution, as it follows single-colony–derived lineages under controlled conditions.

### 2.3 Sanger sequencing

Sanger sequencing protocols were adapted from previous studies (Crossley et al., 2020). The frozen glycerol stocks (−80 °C) were streaked onto charcoal agar plates and incubated at 37 °C for up to 5 days, with plates inspected daily; colonies were harvested once clearly visible. The DNA was extracted and purified using the Bacterial DNA Kit (Omega, D3350) and Genomic DNA Purification Kit (Thermo Fisher, K0512) according to the manufacturer’s protocol. The gene sequences were determined using the ABI 3730XL instrument. Because *B. pertussis* carries three rRNA operons, Sanger traces reflect the consensus sequence and we did not estimate allele frequencies across copies.

### 2.4 Antibiotic MIC measurements

To determine the MIC of the strains, we used E-test strips (Liofilchem) for ERY, ceftriaxone (CRO), sulfamethoxazole (SMX), levofloxacin (LEV), piperacillin (PIP), and sulfamethoxazole-trimethoprim (TMP-SMZ). Briefly, the overnight culture for each strain was diluted to McFarland 0.5 in sterile physiological saline, spread on Bordet–Gengou plates using sterile cotton swabs, and the E-test strips were applied onto the middle of the plates. Plates were incubated at 37 °C and read daily from day 2 onward; MICs were recorded when the inhibition ellipse was clearly defined (typically day 3), with a final read at day 5 if growth was delayed. MIC determinations and downstream phenotypic assays were repeated in independent experiments as described for each assay.

### 2.5 Bacterial relative growth rate measurements

After overnight growth, cultures were diluted in fresh SS broth and adjusted to 0.5 McFarland. OD_600_ was measured every 12 h, and a standard curve was generated. We used *B. pertussis* ATCC 9797 as the control strain to calculate the relative growth rate of all bacterial strains.

### 2.6 Biofilm formation ability and structural analysis by confocal laser scanning microscopy

Biofilm formation was quantified by crystal violet staining in 96-well plates. Overnight cultures were diluted to OD_595_ = 0.1. We added 100 μL of *B. pertussis* culture into 96-well plates, and this was incubated for 96 h at 37 °C. After 24 h, the suspended liquid was removed, cells were washed three times with phosphate-buffered saline (PBS), and fresh SS broth was added every 24 h for a total 96 h incubation period. Afterward, we added 0.1% crystal violet dissolved with absolute ethanol to each well, and we measured the absorbance at OD_595_. To determine the dynamic changes of the biofilm, we repeated the experiment four times, and crystal violet staining was performed at 24, 48, 72, and 96 h.

Structural analysis of *B. pertussis* by confocal laser scanning microscopy (CLSM) was performed according to previously described methods for the cultivation of *B. pertussis* biofilms (Cattelan et al., 2018). The selected dye was SYTO9 (MeilunBio, MC7003).

### 2.7 Virulence factor and resistance-associated antigen/enzyme quantification

We employed an enzyme-linked immunosorbent assay (ELISA) following the manufacturer’s instructions to quantify the expression levels of toxins and the resistance-related, including pertussis toxin (PT^1^), filamentous hemagglutinin (FHA^2^), lipopolysaccharide (LPS^3^), dihydrofolate synthase (DHFS^4^), and dihydrofolate reductase (DHFR^5^). The standard curve for DHFS and DHFR was generated using mouse-derived DHFS and DHFR standards, and the concentrations of DHFS and DHFR in *B. pertussis* were calculated based on this mouse-derived standard curve. To validate the DHFS and DHFR ELISA kits, *B. pertussis* ATCC 9797 (sensitive strain) was treated with sub-MIC concentrations of SMX and TMP (1/2, 1/4, and 1/8 MIC). Cells from overnight cultures were disrupted by ultrasonic lysis, and the clarified supernatant was used for ELISA. For each target, lysates were diluted 1:5, 50 μL was loaded per well, and OD_450_ was measured after HRP development. For each plate, a standard curve was generated using the kit-provided calibrators/standards with known concentrations, and sample concentrations were interpolated from this curve (reported as ng/mL or IU/mL, corrected for the dilution factor). The results for DHFS and DHFR are semi-quantitative and are meant for relative comparison, rather than absolute quantification. All measurements were performed in three independent experiments.

### 2.8 Hemolysis ability

The methods used were adopted from previous reports (Zhou et al., 2021). All the strains were inoculated on charcoal agar plates to reach the stationary growth stage. We inoculated the strains in SS broth, adjusted the OD_600_ to 0.1, and cultivated them at 37 °C for 24 h with shaking at 230 rpm. The complete culture was then diluted to OD_600_ = 0.1 with sterile PBS solution, and the supernatants were collected via filter sterilization. A 100 μL volume of the supernatant was gently added into a tube and mixed with 850 μL of sterile PBS and 50 μL of sheep red blood cells, and incubated at 37 °C for 30 min. The mixtures were centrifuged at 800 × g for 1 min, and the OD of the supernatant was measured at 543 nm. Sterile water and PBS were used as positive and negative controls, respectively. The hemolysis activity was calculated as follows:


$$\begin{array}{l} \text{Hemolysis activity}\;(\%)=\\ \frac{\mathrm{OD}543\;(\text{Sample})-\mathrm{OD}543\;(\text{Negative control})}{\mathrm{OD}543\;(\text{Positive control})-\mathrm{OD}543\;(\text{Negative control})} \end{array}$$

### 2.9 Serum-resistance measurements

We collected serum from healthy volunteers and measured anti-PT IgG by ELISA. Only sera with anti-PT IgG below the assay cut-off (within the kit-defined negative range) were used to minimize potential confounding from recent pertussis exposure; aliquots were stored at −80 °C until use. The ELISA kit was purchased from EUROIMMUN. Before the experiment, we placed a portion of the serum in a water bath at a constant temperature of 56 °C for 30 min to inactivate complement. After centrifuging the overnight cultured bacterial suspensions, the bacterial pellets were washed twice with sterile saline, and the concentration of the bacterial suspensions was adjusted to 1 × 10^6^ CFU/mL with fresh SS broth. We withdrew 25 μL of the bacterial suspension and added it to an Eppendorf tube containing 75 μL of heat-inactivated serum and another with 75 μL of serum. These were mixed well and incubated at 37 °C. At 2, 4, and 6 h, we withdrew the corresponding incubation products, performed a gradient dilution, and spread them evenly on charcoal agar plates. Plates were incubated at 37 °C for up to 5 days to allow colony formation from diluted, serum-exposed samples, and CFUs were counted once colonies were visible and countable. The bacterial survival rate was calculated as follows:


$$\text{Survival rate}\;(\%)=\frac{\text{number of bacteria in serum}}{\text{number of bacteria in inactivated serum}}$$

### 2.10 Antimicrobial peptides MIC measurements

Based on the APD3 ID and amino acid sequence presented in Supplementary Table S1, we synthesized three antimicrobial peptides (AMPs), including beta-casein 197, myticalin C6, and FK-16. Then, we prepared AMP solutions with different concentrations using fresh SS broth, with 11 gradients at concentrations of 32, 16, 8, 4, 2, 1, 0.5, 0.25, 0.125, 0.0625, and 0.03125 μg/mL. The three genotypes of the parental and final strains, as well as the corresponding clinical MRBPs, were cultured overnight, and the bacterial suspensions were adjusted to a McFarland standard of 0.5. These were inoculated into culture medium with different AMP concentrations, and cultivated at 37 °C for 24 h with shaking at 230 rpm. Afterward, the OD600 was measured to obtain the MIC values of the AMPs.

### 2.11 *In vitro* competition experiment

All strains were grown to an OD_600_ of 0.1 in SS broth. An equal volume (2.5 mL) of the AE strains and parental strains was combined and incubated for 72 h with shaking at 230 rpm. The co-cultured bacterial solution from each 24-h incubation period was collected, diluted, and plated on drug-free and drug-treated plates. At the end of incubation, the plate CFU counts were performed, and competition coefficients were calculated. Each test was repeated three times. The competition coefficient is calculated as follows:


$$\begin{array}{l} \text{Competition coefficient}\;(\%)=\\ \frac{\mathrm{CFU}\;\text{in treated plate}}{\mathrm{CFU}\;\text{in treated plate}-\mathrm{CFU}\;\text{in free plate}} \end{array}$$

### 2.12 Whole-genome sequencing analysis and annotation

The bacteria were cultured, and DNA extraction was performed for Sanger sequencing. Sequencing libraries were created using library-building operations performed with the ALFA-SEQ DNA Library Prep Kit. The library quality was assessed using a Qubit 4.0 Fluorometer (Life Technologies, Grand Island, NY) and a Qsep400 High-Throughput Nucleic Acid Protein Analysis system (Houze Biological Technology Co., Hangzhou, China). The libraries were then sequenced on an Illumina NovaSeq 6000 platform, and 150-bp paired-end reads were generated with an average sequencing depth >50 × and a read mapping rate >95%. After preprocessing the data, we used SPAdes (version 3.13.0) for genome assembly to obtain the complete genome. Accordingly, we analyzed the composition of the genome using PSORTb for annotation against the Kyoto Encyclopedia of Genes and Genomes (KEGG), Antibiotic Resistance Genes Database (CARD), and Virulence Factors of Pathogenic Bacteria (VFDB) databases. We used *B. pertussis* CS (CP002695) as the reference genome.

### 2.13 Comparative genomics

We used NUCmer version 3.1 software (Assemblytics^6^) (parameters: -mum, −maxgap = 500, −mincluster = 100) to align the assembled sequences of the cultured strains with the CP002695 genome sequence. The delta-filter from MUMmer 4.0.0 software was used to filter the results, and single-nucleotide polymorphisms were identified using the show-SNPs tool.

The delta file generated using NUCmer version 3.1 software was used as the input to detect structural variations (parameters: unique sequence length required = 1,000, maximum variant size = 10,000, minimum variant size = 1). We matched the mutation sites with the coding DNA sequence position information to determine the related mutation outcomes.

### 2.14 Copy number variation analysis

Using the FASTA data from the whole-genome assembly, we employed CNVnator to detect genome-wide copy number variations (CNVs) for all strains. This CNV analysis focused on deletions/duplications at the genome scale and was not designed to resolve allele-specific variation among the three rRNA operon copies. Information was collected based on the type of CNV (deletion or duplication), where the chromosome and coordinates were located, and the length of the CNV was measured.

### 2.15 Statistical analysis

All hypotheses were pre-specified as pairwise comparisons to a single reference within each analysis panel. For ELISA readouts (PT, FHA, LPS, DHFS, DHFR), the reference sample was the corresponding parental strain within the same genetic background (M0), and values were plotted as absolute concentrations to this reference unless stated otherwise. Data are presented as mean ± SD. Two-sided Welch’s *t*-tests (unequal variances) were used for all two-group comparisons. For time-course readouts, pairwise Welch tests versus the reference were performed at each time point; for single-timepoint readouts, one test per marker was performed. Family-wise error was controlled within each block of comparisons that shared the same marker and reference using the Holm procedure.

## 3 Results

### 3.1 Resistance outcomes in the three tested genetic backgrounds

At present, the Clinical and Laboratory Standards Institute (CLSI) and European Committee on Antimicrobial Susceptibility Testing (EUCAST) have not provided break-point criteria for *B. pertussis* antimicrobial susceptibility. Because no CLSI/EUCAST breakpoints are available for *B. pertussis*, we used MIC >256 μg/mL as an operational definition of high-level macrolide resistance (MRBP) in this study. In our dataset, parental strains had MIC <0.016 μg/mL, whereas clinical MRBPs had MIC >256 μg/mL.

**Figure 2:**
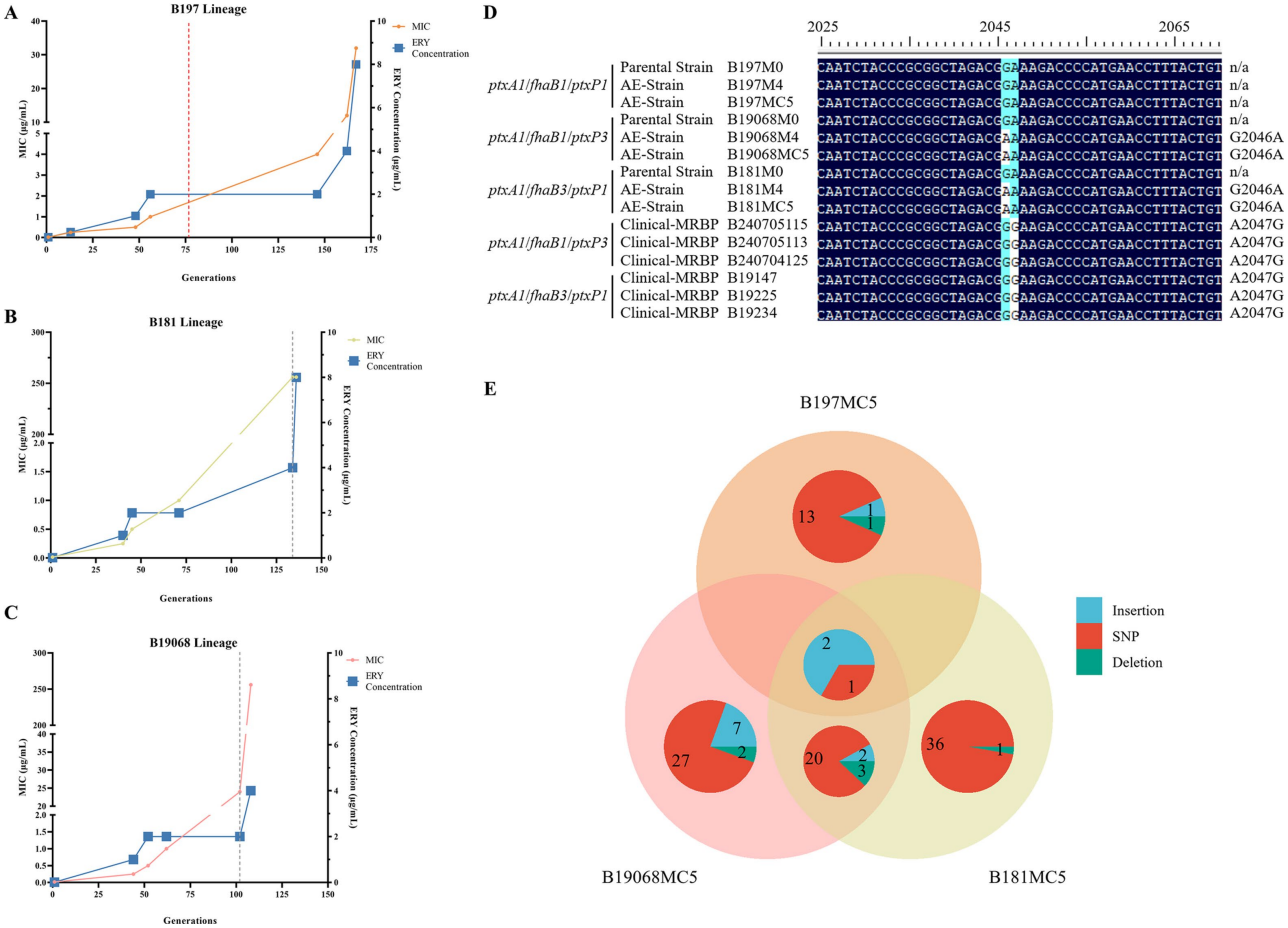
Change in macrolide resistance of AE strains. **(A)** The MIC and erythromycin concentration of the B197 lineage; the red dashed lines indicate the first detection of efflux-associated variants. **(B)** The MIC and erythromycin concentration of the B181 lineage; the gray dashed lines indicate the first detection of the 23S rRNA G2046A substitution in the consensus sequence. **(C)** The MIC and erythromycin concentration of the B19068 lineage; the gray dashed line indicates the first detection of the 23S rRNA G2046A substitution. **(D)** The region of erythromycin target with AE strains and clinical MRBP. **(E)** The mutation types in the three lineages. AE, Antibiotic-exposed; MIC, minimum inhibitory concentration; MRBP, macrolide-resistant *Bordetella pertussis*. Each generation on plates containing graded erythromycin concentrations.

Based on the ERY MIC values, we prepared charcoal agar plates containing the four concentrations of ERY. Single colonies from the parental strains were inoculated onto the plates, and we used the E-test to determine the MIC values of each generation of bacteria (Figures 2A–C). For this stepwise erythromycin induction experiment, three parental strains were used, representing three distinct genetic backgrounds. All three lineages increased to 1 μg/mL rapidly, then remained at 2 μg/mL for prolonged passages before growth at 4 μg/mL emerged. The ERY MIC of B197, B19068, and B181 remained at 2 μg/mL for 107, 50, and 89 generations, respectively, based on MIC measurements at each generation on plates containing graded erythromycin concentrations. B19068 and B181 reached high-level ERY resistance (MIC >256 μg/mL), similar to the high-level resistance observed in the clinical MRBP comparator strains. In contrast, B197 acquired growth on 4 μg/mL ERY at a later stage, but its ERY MIC plateaued at 32 μg/mL, indicating reduced susceptibility but not high-level resistance under the tested conditions. In the B181 and B19068 induction series, the final AE strains developed high-level resistance and carried the 23S rRNA G2046A mutation in the consensus sequence, whereas the clinically predominant A2047G mutation was not detected in these lineages. Moreover, no 23S rRNA mutation was detected in the B197 lineage (Figure 2D). With only one induction series per background, the timing and duration of MIC shifts in Figure 2 may be sensitive to stochastic variation.

### 3.2 Impact of resistance-induced mutations on proliferation, biofilm formation, and virulence

**Figure 3:**
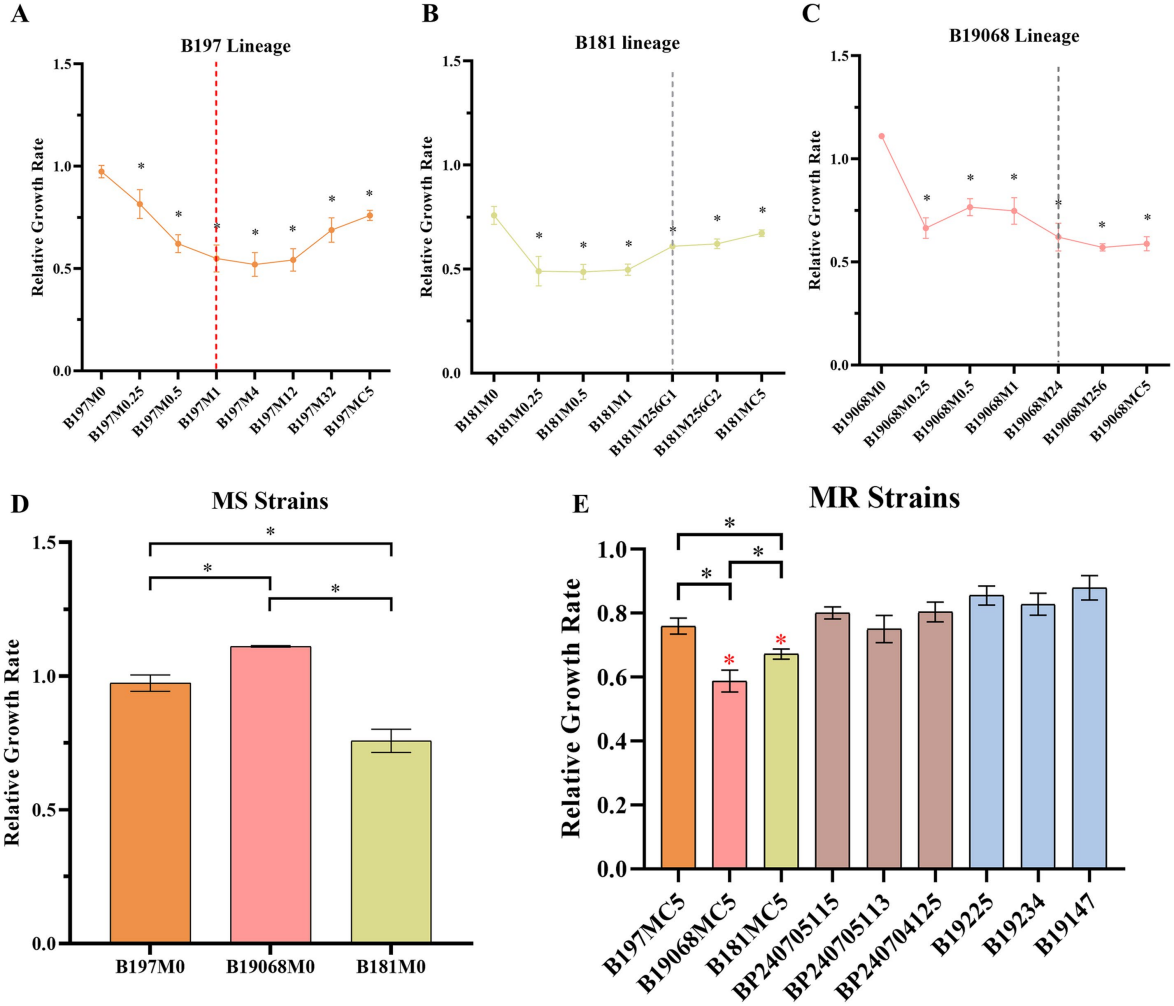
Relative growth rate of all strains. *Bordetella pertussis* ATCC 9797 was used as the reference strain. **(A)** Relative growth rate of the B197 lineage; the red dashed lines indicate the first detection of efflux-associated variants. **(B)** Relative growth rate of the B181 lineage; the gray dashed lines indicate the first detection of the 23S rRNA G2046A substitution. **(C)** Relative growth rate of the B19068 lineage; the gray dashed lines indicate the first detection of the 23S rRNA G2046A substitution. The black * in **(A–C)** represent the comparison of the strain with its corresponding parental strain (*p*-value < 0.05). **(D)** Relative growth rate of macrolide-sensitive *B. pertussis*. **(E)** Relative growth rate of three macrolide-resistant *B. pertussis* and clinical macrolide-resistant *B. pertussis*. The red * represents the comparison of strains with the clinical strains (*p*-value < 0.05). Each experiment was repeated three times. MR, macrolide-resistant; MS, macrolide-sensitive.

**Figure 4:**
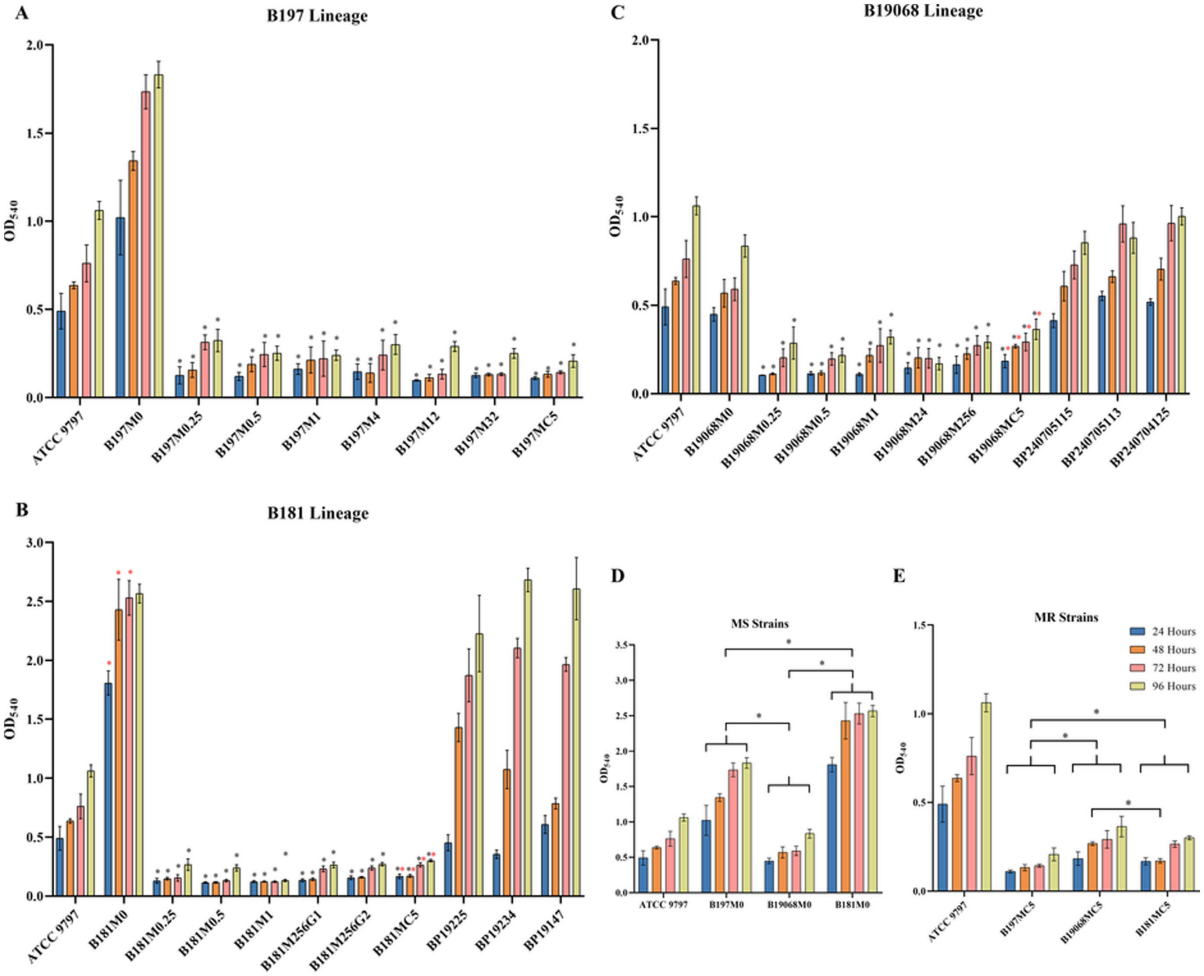
Biofilm formation ability of all strains. **(A)** The biofilm formation ability of the B197 lineage. **(B)** The biofilm formation ability of the B181 lineage and clinical MRBP. **(C)** The biofilm formation ability of the B19068 lineage and clinical MRBP. The black * in **(A–C)** represent the comparison of the strain with its corresponding parental strain. The red * in **(A–C)** represent the comparison of strains with the clinical strains. **(D)** The biofilm formation ability of macrolide-sensitive *B. pertussis*. **(E)** The biofilm formation ability of final AE and clinical MRBP. The * indicates significance with *p* < 0.05. Each experiment was repeated four times. AE, Antibiotic-exposed; MRBP, macrolide-resistant *Bordetella pertussis.*

**Figure 5:**
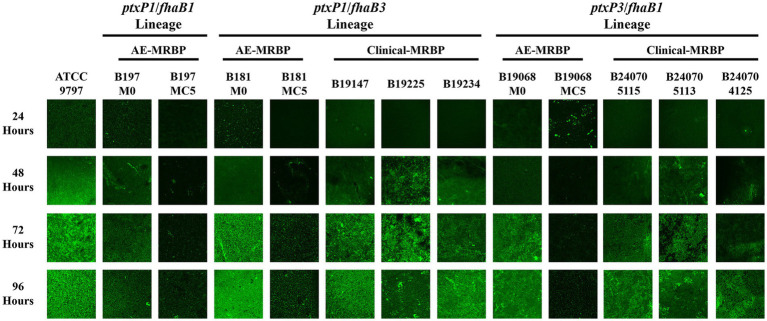
The 96-h biofilm formation ability of all strains through confocal laser scanning microscopy (CLSM). AE, Antibiotic-exposed; MRBP, macrolide-resistant *Bordetella pertussis*.

Proliferation and biofilm formation of *B. pertussis* were reduced in the AE strains during stepwise ERY induction. We compared the parental strains (M0) and final strains with the corresponding clinical MRBPs. *B. pertussis* ATCC 9797 was used as the reference strain. The relative growth rates are presented in Figure 3, and growth decreased across the three lineages during the induction series. The B197 lineages showed a marked decrease in growth rate from the generation in which efflux-associated mutations appeared (B197M1, B197M4, and B197M12). By the final generation (B197MC5), their growth rate recovered and became the highest among the three lineages. Moreover, as shown in Figure 3E, the final AE strains of B19068 and B181 grew significantly more slowly than the corresponding clinical MRBPs, which were selected to share the same *fhaB* and *ptxP* allelic backgrounds as the parental strains. We further examined biofilm formation among the parental strains, where B19068 exhibited the weakest ability and B181 the strongest (Figure 4). During the induction series, biofilm formation of all AE strains decreased, and the biomass of the final strains at 96 h was significantly lower than that of the parental strains at 96 h. Moreover, biofilm formation of the parental strains of B19068 and B181 was higher than their clinical MRBPs before 72 h, whereas B19068MC5 and B181MC5 formed significantly less biofilm than the corresponding clinical MRBPs under the *in vitro* conditions tested. CLSM showed that the parental strains formed the strongest biofilms, while the AE MRBPs formed the weakest (Figure 5). The discrepancies between OD-based measurements and the fluorescent signal in CLSM images may reflect differences in biofilm structure, staining efficiency, or cell viability, rather than absolute differences in biomass.

**Figure 6:**
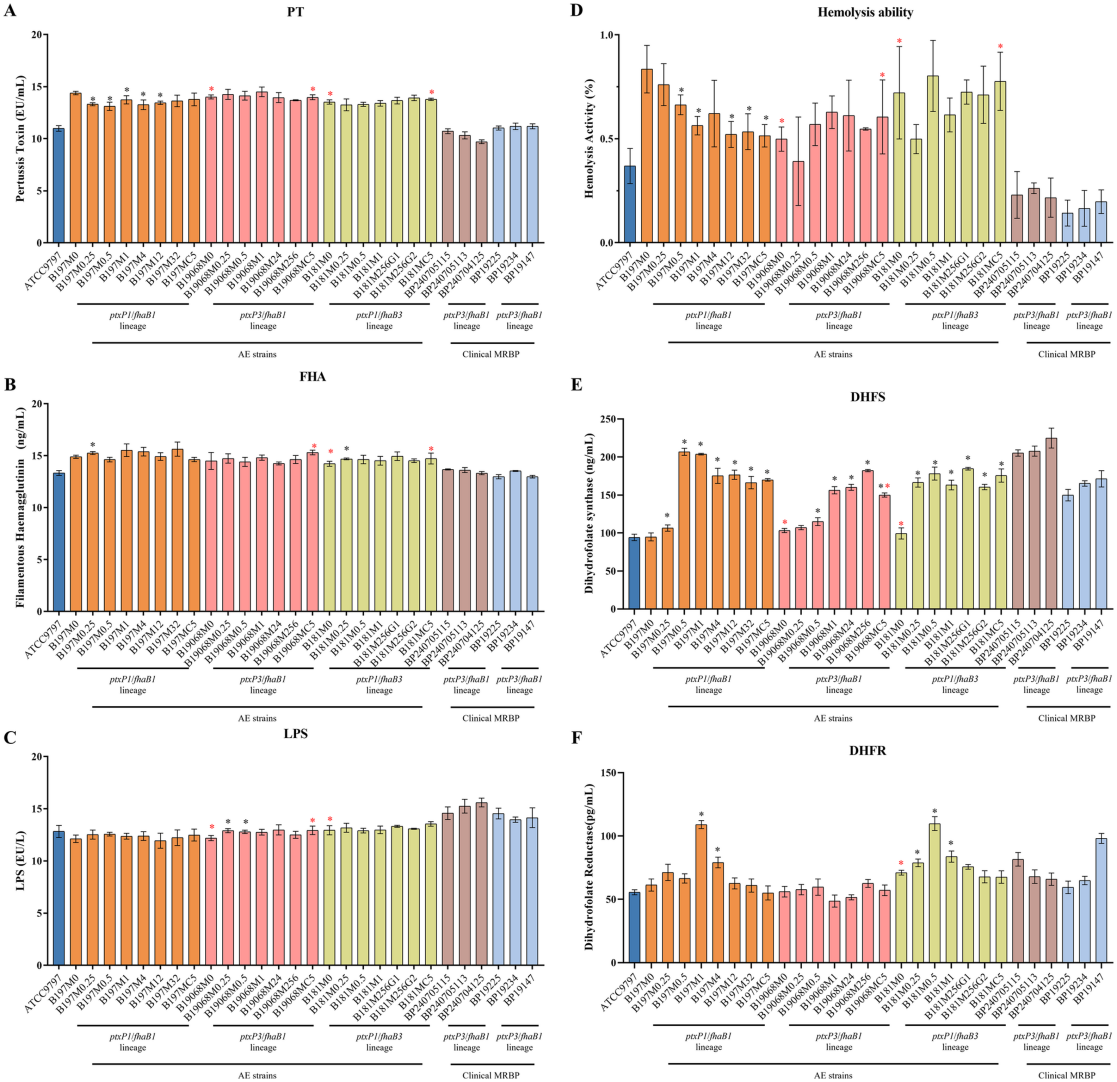
Virulence factor and resistance-associated antigen/enzyme quantification across strains. **(A)** Pertussis toxin (PT); **(B)** filamentous hemagglutinin (FHA); **(C)** lipopolysaccharide (LPS); **(D)** hemolytic activity; **(E)** dihydrofolate synthase (DHFS); and **(F)** dihydrofolate reductase (DHFR). ELISA concentrations were calculated from kit-provided standard curves and are reported as absolute concentrations; values were corrected for the 1:5 dilution as described in Methods. Black asterisks indicate comparisons between each induced isolate and its corresponding parental strain (M0) within the same genetic background. Red asterisks indicate comparisons between indicated isolates and the designated clinical reference strain(s) within the same background. Statistical tests are described in Statistical analysis; *p < 0.05. Data represent three independent experiments. AE, Antibiotic-exposed; MRBP, macrolide-resistant *Bordetella pertussis*.

Moreover, we quantified PT, FHA, and LPS levels by ELISA (Figures 6A–C) and assessed hemolytic activity across the parental strains and AE lineages. ELISA values are reported as absolute concentrations interpolated from kit-based standard curves and corrected for dilution, representing toxin/antigen-associated readouts under defined *in vitro* conditions.

For PT, within the B197 lineage, EU/mL values from B197M0.25 through B197M12 were lower than the parental strain, whereas the final AE strains (B197MC5) returned to levels similar to the parental strain, possibly reflecting a transient reduction in toxin production during intermediate generations. In the B19068 and B181 lineages, PT levels showed minor fluctuations across all generations, with no consistent change relative to the parental strains.

FHA concentrations remained largely stable across all lineages; compared with the corresponding clinical MRBPs, FHA was somewhat higher in B19068MC5, B181M0, and B181MC5. Similarly, LPS concentrations remained largely stable throughout the induction series, although some minor variations were observed among generations and lineages, with certain B19068 lineages showing slightly higher values than their parental strains.

As shown in Figure 6D, hemolytic activity in B197 lineage tended to decrease, whereas in the B19068 and B181 lineages, activity varied without a consistent trend. The final AE strains exhibited hemolytic activity similar to their parental strains. Overall, hemolytic activity of the AE lineages was generally higher than that of the clinical MRBPs.

### 3.3 Altered sulfonamide susceptibility is associated with metabolic readouts during induction

To explore whether the induction of macrolide resistance leads to changes in resistance to other antibiotics as a result of various mutations, four families (fluoroquinolones, cephalosporins, sulfonamides, and β-lactam antibiotics) were evaluated (Table 2). These antibiotics are used in the clinical treatment of whooping cough, often as alternatives to macrolides, which are the first-line antibiotics for the empirical treatment of whooping cough. In representative generations of each lineage, MIC values for LEV, CRO, and PIP ranged from 0.004 to 0.032 μg/mL, 0.047 to 1 μg/mL, and below the detection limit (<0.016 μg/mL), respectively.

**Table 2:** Minimum inhibitory concentration (MIC) of other relative antibiotics.

Genotype	Strains	LEV	CRO	PIP	SMX	TMP-SMZ
*ptxA1*/*fhaB1*/*ptxP1*	ATCC9797	0.006	0.047	<0.016	0.38	0.047
*ptxA1*/*fhaB1*/*ptxP1*	B197M0	0.006	0.125	0.094	1	0.125
	B197M0.25	0.006	0.125	<0.016	256	0.75
	B197M0.5	0.006	0.125	<0.016	1,024	>32
	B197M1	0.012	0.125	<0.016	1,024	>32
	B197M4	0.012	0.5	<0.016	1,024	>32
	B197M12	0.012	0.5	<0.016	1,024	>32
	B197M32	0.016	0.5	<0.016	1,024	6
	B197MC5	0.016	1	0.047	1,024	0.5
*ptxA1*/*fhaB1*/*ptxP3*	B19068M0	0.006	0.094	<0.016	1.5	0.5
	B19068M0.25	0.006	0.094	<0.016	24	0.75
	B19068M0.5	0.006	0.094	0.032	512	1.5
	B19068M1	0.004	0.125	0.032	1,024	1.5
	B19068M24	0.004	0.19	0.032	1,024	1.5
	B19068M256	0.004	0.19	0.032	1,024	0.5
	B19068MC5	0.004	0.19	0.032	1,024	0.5
*ptxA1*/*fhaB3*/*ptxP1*	B181M0	0.004	0.064	<0.016	0.019	0.75
	B181M0.25	0.006	0.064	0.032	1,024	1.5
	B181M0.5	0.006	0.125	0.023	1,024	4
	B181M1	0.006	0.125	0.023	1,024	>32
	B181M256G1	0.006	0.125	0.016	1,024	4
	B181M256G2	0.006	0.064	<0.016	1,024	4
	B181MC5	0.004	0.047	<0.016	1,024	0.5
*ptxA1*/*fhaB1*/*ptxP3*	BP240705115	0.004	0.064	<0.016	1,024	0.5
	BP240705113	0.006	0.032	<0.016	1,024	0.5
	BP240704125	0.006	0.064	<0.016	1,024	0.5
*ptxA1*/*fhaB3*/*ptxP1*	BP19225	0.004	0.047	<0.016	0.5	0.38
	BP19234	0.032	0.047	<0.016	1	0.25
	BP19147	0.006	0.047	<0.016	1	0.125

*LEV, Levofloxacin; CRO, Ceftriaxone; PIP, Pipericillin; SMX, Sulfamethoxazole; TMP-SMZ, Trimethoprim-Sulfamethoxazole.

Sulfonamides are among the main alternative antibiotics as proposed by the World Health Organization and medical institutions worldwide (Tiwari et al., 2005). Therefore, we tested SMX and TMP-SMZ to characterize changes in sulfonamide susceptibility during induction across the three tested genetic backgrounds. For SMX, resistance emerged early during induction, and high-level resistance was reached before the bottleneck phase (2–4 μg/mL) with MIC >1,024 μg/mL. No mutations related to sulfonamide resistance (*sul1*, *sul2*, etc.) were detected in these lineages, and no variants were identified in DHFS-associated genes (*folC*, *folA*). However, as shown in Figure 6E, there was a trend of DHFS overexpression in the strains, which reached its highest level when the SMX MIC was >1,024 μg/mL. Among the clinical isolates included here, macrolide-resistant *ptxP3*/*fhaB1* strains showed higher DHFS readouts than the *ptxP1*/*fhaB3* strains tested, which remained sulfonamide-susceptible under our assay conditions. This observation is consistent with background-associated differences in metabolic readouts, although causality and the responsible loci cannot be assigned in this dataset. For TMP-SMZ, MICs increased and then decreased across all lineages. Although CLSI and EUCAST have not defined a cut-off for sulfonamide resistance in *B. pertussis*, the MIC of the B197 and B181 lineages exhibited resistance at levels of > 32 μg/mL in the process, which eventually decreased to 0.5 μg/mL. The B19068 lineage differed slightly but also showed an increasing then a decreasing trend during the process. In addition to increased DHFS readouts, DHFR readouts also increased during induction in the B197 and B181 lineages (Figure 6F). B19068 showed a non-significant increase in DHFR expression levels, with the highest expression observed in B19068M0.5 when the MIC reached 1.5 μg/mL. In some lineages, peaks in DHFS/DHFR expression coincided with periods of increased TMP-SMZ MIC, although this pattern was not consistent across all generations or backgrounds. These observations suggest that metabolic adjustment may contribute to short-term sulfonamide-related phenotypes in the absence of detectable gene mutations, without implying a strict correlation between DHFR/DHFS readouts and MIC. Similar sulfonamide-related changes were observed in all three tested backgrounds under the present experimental conditions. To ensure the validity of the DHFS and DHFR ELISA kits, *B. pertussis* ATCC 9797 (sensitive strain) was treated with sub-MIC concentrations of SMX and TMP (1/2, 1/4, and 1/8 MIC). As shown in Supplementary Table S2, both DHFS and DHFR readouts showed a consistent downward trend under sub-MIC SMX and TMP exposure.

### 3.4 Serum survival, antimicrobial peptide susceptibility, and *in vitro* competitive fitness

In natural settings, bacteria encounter complex conditions. We first tested serum survival of the AE and clinical strains. Heat treatment at 56 °C for 30 min inactivated complement in serum, thereby limiting complement-mediated killing (Morgan and Harris, 2015). As shown in Table 3, the MSBPs did not survive in serum, and serum survival was not observed in the AE strains during the induction series. In contrast, clinical MRBPs, regardless of genotype, showed measurable serum survival. In parallel, heat-inactivated serum was used to confirm complement-dependent killing.

**Table 3:** The serum-resistance of all strains.

Strains	Time (hour)		
	2	4	6
ATCC9797	n/a	n/a	n/a
B197M0	n/a	n/a	n/a
B197M0.25	n/a	n/a	n/a
B197M0.5	n/a	n/a	n/a
B197M1	n/a	n/a	n/a
B197M4	n/a	n/a	n/a
B197M12	n/a	n/a	n/a
B197M32	n/a	n/a	n/a
B197MC5	n/a	n/a	n/a
B19068M0	n/a	n/a	n/a
B19068M0.25	n/a	n/a	n/a
B19068M0.5	n/a	n/a	n/a
B19068M1	n/a	n/a	n/a
B19068M24	n/a	n/a	n/a
B19068M256	n/a	n/a	n/a
B19068MC5	n/a	n/a	n/a
B181M0	n/a	n/a	n/a
B181M0.25	n/a	n/a	n/a
B181M0.5	n/a	n/a	n/a
B181M1	n/a	n/a	n/a
B181M256G1	n/a	n/a	n/a
B181M256G2	n/a	n/a	n/a
B181MC5	n/a	n/a	n/a
BP240705115	90%	60%	n/a
BP240705113	87%	58%	n/a
BP240704125	89%	21%	n/a
BP19225	71%	2%	n/a
BP19234	10%	6%	n/a
BP19147	14%	9%	2%

n/a: After treated with non-inactivated serum, the bacterial colonies number was 0 CFU/mL.

Bacteria are also exposed to antimicrobial peptides (AMPs). We selected two human AMPs (beta-casein 197 and FK-16) and one AMP from Mytilus spp. for MIC testing (Table 4). Beta-casein 197 in human breast milk may contribute to protection against *B. pertussis*, and FK-16 is an active fragment of LL-37. Both parental strains showed resistance to beta-casein 197. The AE MRBPs showed markedly reduced MICs to beta-casein 197 and FK-16 (MIC < 0.03125 μg/mL), whereas the clinical MRBPs retained partial resistance. Myticalin C6, an AMP derived from marine organisms, is not commonly encountered by *B. pertussis* and has broad antimicrobial activity (Leoni et al., 2017). The MSBPs and clinical MRBPs have lower resistance to myticalin C6, compared with that to beta-casein 197 and FK-16, with only some strains exhibiting resistance. Similarly, the AE MRBPs lost full resistance.

**Table 4:** The antibacterial peptide minimum inhibitory concentration (MIC).

Strains	Beta-Casein 197	FK-16	Myticalin C6
ATCC9797	0.03125	<0.03125	<0.03125
B197M0	0.0625	0.03125	<0.03125
B197MC5	<0.03125	<0.03125	<0.03125
B19068M0	0.0625	0.03125	0.03125
B19068MC5	<0.03125	<0.03125	<0.03125
B181M0	0.03125	<0.03125	0.03125
B181MC5	<0.03125	<0.03125	<0.03125
BP240705115	<0.03125	0.25	<0.03125
BP240705113	0.25	0.03125	0.03125
BP240704125	<0.03125	<0.03125	0.0625
BP19225	0.0625	0.125	<0.03125
BP19234	0.0625	0.0625	<0.03125
BP19147	0.0625	0.0625	0.125

In addition, *in vitro* competition experiments (Table 5) indicated weak competitive ability for the three lineages at early and later stages, and most AE strains showed poor competitive ability; the competitive abilities of the parental strains were strongly inhibited under the tested conditions.

**Table 5:** The competition coefficient of antibiotic-exposed (AE) strains.

Strains	Co-culture time (hour)		
	24	48	72
B197M0.25	2.1%	9.6%	9.2%
B197M0.5	4.3%	n/a	n/a
B197M1	3.9%	n/a	n/a
B197M4	n/a	n/a	n/a
B197M12	n/a	n/a	n/a
B197M32	n/a	n/a	n/a
B197MC5	0.2%	n/a	n/a
B19068M0.25	10.6%	10.1%	8.5%
B19068M0.5	9.4%	6.0%	n/a
B19068M1	6.8%	5.3%	n/a
B19068M24	n/a	n/a	n/a
B19068M256	n/a	n/a	n/a
B19068MC5	0.1%	n/a	n/a
B181M0.25	6.1%	5.5%	4.9%
B181M0.5	2.3%	n/a	n/a
B181M1	n/a	n/a	n/a
B181M256G1	n/a	n/a	n/a
B181M256G2	n/a	n/a	n/a
B181MC5	0.4%	0.2%	n/a

Competition coefficient = AE-strain/parental-strain*100%, n/a: the CFU/mL of AE strains were 0.

### 3.5 Resistance-linked mutation profiles and conserved copy-number variants

Based on the CP002695 reference genome, the mutation sites of the three lineages were annotated and compared using the CARD database, and CNVs were analyzed separately. The WGS results of the B19068 and B181 lineages, representing two dominant clinical-MRBP genetic backgrounds, were consistent with the Sanger sequencing results, with 23S rRNA G2046A as the only detected macrolide resistance–associated variant. In contrast, no mutations or CNVs were detected in sulfonamide resistance–related genes in the B197 lineage, whereas mutations were observed in three efflux-associated loci (Supplementary Table S3), which may be associated with the observed lower MIC to macrolides; the genetic basis of the sulfonamide phenotype cannot be inferred from these data.

The number and types of mutations in each AE strain are shown in Figure 2E (excluding unknown and hypothetical proteins). The three lineages had three common mutations, including two insertions and one gene variant (Supplementary Table S4). The two insertions were located in the L4 and L22 segments of the 50S ribosomal protein. Moreover, the B197 lineage shared these three mutations with the B19068 and B181 lineages. Alternatively, the B19068 and B181 lineages had relatively more mutations, including 21 gene variants, 4 insertions, and 3 deletions (Supplementary Table S5). Mutations were mainly concentrated in the encoding genes of 50S ribosomal proteins, pertactin precursor, filamentous hemagglutinin, and fimbriae. Each of the three lineages harbored many unique mutations; in B197, they were concentrated in metabolism-related genes, such as *cusC*, *talB*, and *rpoC* (Supplementary Table S3). In addition, the *rpoC* mutation is often considered a compensatory mutation that maintains bacterial fitness in *Mycobacterium tuberculosis* (Comas et al., 2011). This observation is consistent with a possible compensatory role under the tested conditions. The unique mutations of B181 involve a resistance-related gene *rplD* and a toxin-related gene *fhaB*, with the rest mainly affecting bacterial metabolism genes, such as *hrcA*, *sphB2*, and *maeB* (Supplementary Table S6). Compared to the B181 and B197 lineages, B19068 had more mutations that occurred in the encoding genes of virulence proteins, such as *prn*, *fliM*, *fhaS*, and *ptxC*, in addition to many metabolism-related mutations, such as *ureA* and *wcbA* (Supplementary Table S7). The three lineages had few identical site mutations and mutated genes. Compared to the clinical strains, the AE strains had only a few mutations, which were mainly concentrated in bacterial chemotaxis- and metabolism-related genes (Supplementary Tables S8, S9). Moreover, although some AE strains carried variants in PT- or FHA-associated loci, we did not observe a corresponding, consistent shift in the PT/FHA ELISA readouts under the assay conditions used.

CNVs were analyzed for all strains against the CP002695 reference genome, because repeated laboratory passaging may be accompanied by structural changes beyond SNPs/indels. Across the induced isolates, CNV analysis identified two deletions that were conserved in the dataset (971501–995500 and 1222501–1229500; Supplementary Table S10). Moreover, the disappearance of duplication of the M0.25 generations co-occurred with the reduction of biofilm formation and growth ability. In addition, CNVs of the three parental and final strains also exhibited significant differences, although most of the genes were focused on the synthesis, metabolism, and transport of bacterial physiological functions.

## 4 Discussion

Bacterial resistance under antibiotic pressure can involve distinct mutations, potentially influenced by genetic and environmental constraints (Baquero et al., 2021). Using three representative clinical genetic backgrounds, our stepwise erythromycin induction experiment showed that distinct resistance-associated variants, resistance trajectories, and accompanying phenotypic changes can emerge under the same ERY exposure regimen. In addition to macrolide resistance, concurrent changes in sulfonamide susceptibility were observed, together with altered DHFS/DHFR-associated readouts, suggesting potential metabolic adjustments during induction. Antibiotic-exposed strains also exhibited reduced growth and biofilm formation, consistent with possible fitness trade-offs under these conditions. Furthermore, susceptibility to antimicrobial peptides varied across lineages, indicating that these phenotypes may be condition-dependent and influenced by the experimental context.

Although *B. pertussis* is often described as genetically conserved, clinically circulating isolates sharing *ptxP*/*fhaB* allele labels may still differ at multiple loci across the genome. In the framework proposed by Bridel et al., the parental isolates analyzed here can be contextualized within commonly reported lineage IIb-related backgrounds, including *ptxP3*/*fhaB1*- and *ptxP1*-associated lineages circulating in China and elsewhere (Bridel et al., 2022). In our result, the three parental backgrounds showed differences in the resistance-associated variants recovered under stepwise ERY induction and in several phenotypic readouts. These findings support the use of *ptxP*/*fhaB* allele combinations as practical markers of broader genetic background in this experimental setting.

In our study, two lineages acquired the canonical macrolide-resistance mutation 23S rRNA G2046A and reached high-level ERY resistance (MIC >256 μg/mL). In contrast, the B197 series followed an efflux-associated route and plateaued at a lower MIC (32 μg/mL). Together, these results illustrate divergent resistance trajectories observed across genetic backgrounds under the experimental conditions used, which can be resolved by genomic screening. Consistent with a trade-off, we observed reduced growth and biofilm formation in antibiotic-exposed strains. Prior work has suggested that epistasis may influence antibiotic-resistance evolution (Jochumsen et al., 2016; Lukacisinova et al., 2020). In our data, CNV patterns differed among the parental backgrounds, and the AE MRBP lineages B19068 and B181 showed CNV profiles that differed from those of B197. Whether these differences contribute to the observed phenotypes will require further validation.

In this induction system, prolonged exposure to sublethal concentrations of ERY (2–4 μg/mL) was associated with the selection of resistance mutations. The extended stagnation at 2–4 μg/mL (Figure 2) may reflect constraints on the available resistance routes under this regimen; however, with a single induction series per background, the timing of these transitions should be interpreted cautiously. In the present experiment, the B19068 and B181 induction series reached high-level resistance together with a 23S rRNA G2046A substitution in the consensus sequence, whereas B197 accumulated efflux-associated variants and plateaued at a lower MIC. This contrast suggests that distinct resistance trajectories can arise in different tested backgrounds under the same induction regimen.

Clinical *B. pertussis* strains experience pressures beyond antibiotics and may therefore differ from antibiotic-exposed isolates. For example, the complement system in human serum exerts pressure on bacteria, and various AMPs inhibit bacteria; these conditions were not present in the AE strains (Morgan and Harris, 2015). Clinical MRBPs possess serum resistance, which is in contrast to the MSBPs. Consistent with the absence of complement pressure during *in vitro* induction, serum survival was not observed in MSBPs or AE strains. As this characteristic is lacking in the parental strains, it will not be gained without the corresponding pressures. In contrast to laboratory-selected strains, clinical *B. pertussis* strains acquire resistance mutations through continuous antibiotic pressure and experience additional selective pressures, such as host immune responses. After induction, MICs to beta-casein 197 and FK-16 decreased markedly in the AE strains. Moreover, most strains’ myticalin C6 resistance levels were lower than those of the two human AMPs. This influence was particularly prominent in long term experimental evolution, which was similar to that of *E. coli*, which was studied for 1,000 generations in either maltose- or glucose-limited media. As a result, small changes in the environment can profoundly affect the adaptation and divergence (Yona et al., 2018). Accordingly, resistant strains induced under a single laboratory environment may differ substantially from evolutionary trajectories occurring in natural host settings (Maddamsetti et al., 2017). Overall, our serial passaging under simplified laboratory conditions revealed genomic changes beyond the intended resistance-associated variants, including structural variation and deletions in some AE strains; therefore, AE strains should be interpreted as regimen-specific outcomes that may not fully reflect processes occurring during clinical infection.

Bacteria develop resistance through a complex interplay of mutations that affect their growth, biofilm formation, and other fitness traits (Ferenci, 2016). Although *ptxP3* lineages are often reported to produce more PT than *ptxP1* lineages, we did not observe a clear *ptxP3*–*ptxP1* separation in our PT ELISA readouts under the *in vitro* conditions used. This could be due to growth-phase/culture dependence of PT production, limited sampling across backgrounds, and genome-wide background effects beyond *ptxP* that may mask genotype-level trends. Therefore, we interpret these PT data as assay-specific rather than as evidence against the population-level association. In contrast, the clinical MRBPs, which are exposed to a range of environmental pressures, have evolved strategies that balance resistance with maintaining competitive traits such as strong growth and biofilm formation. The low virulence of clinical MRBPs may lead to prolonged clinical visits and increased transmission opportunities, while their robust growth and biofilm-forming abilities enhance their competitiveness in natural settings (Fu et al., 2024). Interestingly, the AE strains, which were cultured under simplified laboratory conditions, did not face the same competitive pressures and thus did not undergo the same changes in virulence or other fitness-related traits. For example, mutations in core genes such as *rpoA* and *rpoC*, which rarely appear in clinical resistant strains, were present in the AE strains, reflecting the differences in selective pressures between laboratory and natural environments (Long et al., 2015).

Cross-resistance dynamics were also observed in this study, where resistance to one antibiotic can lead to reduced susceptibility to others, which has significant implications for treatment strategies (Imamovic and Sommer, 2013). During the process, AE strains exhibited low-level increased resistance to some antibiotics (e.g., LEV, CRO, and PIP) and marked shifts in sulfonamide susceptibility. Changes in the level of resistance to other antibiotics underscore the dynamic nature of bacterial fitness. In the treatment of whooping cough, sulfonamides have been used as alternatives to macrolides when initial treatment fails (Tiwari et al., 2005). In our study, the AE strains exhibited metabolic readouts consistent with metabolic compensation, similar to that reported in *Group A Streptococcus* through the overexpression of DHFS and DHFR, which may change the resistance level of TMP-SMZ and SMX, even in the absence of mutations and CNVs in the SMX- and TMP-related resistant genes (Rodrigo et al., 2022; Kneis et al., 2023). In our study, the AE MRBPs showed a high level of resistance to TMP-SMZ compared to the clinical MRBPs, which may be associated with continuous ERY exposure under the induction regimen. These observations raise the possibility that prior macrolide exposure could influence sulfonamide-related phenotypes under some conditions; however, this remains to be validated in clinical settings. These findings suggest that TMP-SMZ-related phenotypes may be context-dependent after prior macrolide exposure under experimental conditions, although their clinical relevance remains to be determined.

The reduced resistance of AE strains to host defense mechanisms, evidenced by the loss of serum resistance and increased susceptibility to human antimicrobial peptides (FK-16 and beta-casein 197), highlights the limitations of simplified experimental conditions. Our results indicate that macrolide selection under simplified laboratory conditions was not sufficient to recapitulate host-associated immune-evasion phenotypes commonly observed in clinical MRBPs. AE strains did not show increased serum resistance (Table 3), suggesting that macrolide resistance and serum resistance are not obligatorily linked and may require additional host-derived pressures (e.g., ERY plus serum/complement passaging) to co-evolve. Key divergences included reduced growth/biofilm formation and increased AMP susceptibility in AE strains, despite similar toxin readouts compared with clinical isolates. These findings highlight that laboratory passaging may not capture host-driven selection on immune-evasion and fitness traits.

Our study was conducted under simplified *in vitro* conditions, limiting direct extrapolation to host settings; future work should test whether the observed variants and phenotypes persist under more host-relevant pressures (e.g., respiratory epithelial co-culture or antimicrobial components). Several intergenic/unknown variants (Supplementary Table S11) may reflect regulatory effects, but functional relevance was not assessed and will require targeted validation. We also lacked no-antibiotic passaging controls, so regimen-associated changes could not be fully separated from passaging adaptation. Accordingly, the present dataset does not permit locus-specific conclusions about the mutational route and does not resolve whether the observed sulfonamide-related changes are reproducibly shared across backgrounds. Finally, we did not resolve allele-specific variation among the three rRNA operon copies, and thus could not infer fixation of 23S rRNA substitutions across copies.

## 5 Conclusion

Our study establishes a controlled induction framework in *Bordetella pertussis* and shows that stepwise erythromycin exposure can be accompanied by divergent MIC trajectories, resistance-associated variants, and phenotypic changes across three clinical genetic backgrounds. In two backgrounds, high-level ERY resistance was accompanied by a 23S rRNA G2046A substitution in the consensus sequence, whereas the third background followed a lower-MIC trajectory with efflux-associated variants. Together, these findings provide a basis for future validation in replicated evolution experiments and more host-relevant models.

## Data Availability

The datasets presented in this study can be found in online repositories. The names of the repository/repositories and accession number(s) can be found below: https://www.ncbi.nlm.nih.gov/genbank/, BioProject ID: PRJNA1276999.

## Footnotes

1
http://www.shmabio.com/products/show36206098.html

2
http://www.shmabio.com/products/show36206107.html

3
http://www.mmbio.cn/goods.php?id=28440

4
http://www.mmbio.cn/goods.php?id=30484

5
http://www.mmbio.cn/goods.php?id=30482

6 http://assemblytics.com/ and https://github.com/marianattestad/assemblytics

## Supplementary material

SUPPLEMENTARY FIGURE S1 Raw OD600 growth curves of antibiotic-exposed lineages from the three genetic backgrounds.
