# Supplementary figures and images for: Macrolide resistance trajectories across three Bordetella pertussis genetic backgrounds under stepwise erythromycin exposure
Source: Front Microbiol. 2026 Apr 17;17:1803864. doi: 10.3389/fmicb.2026.1803864 (PMC13133076; doi:10.3389/fmicb.2026.1803864)

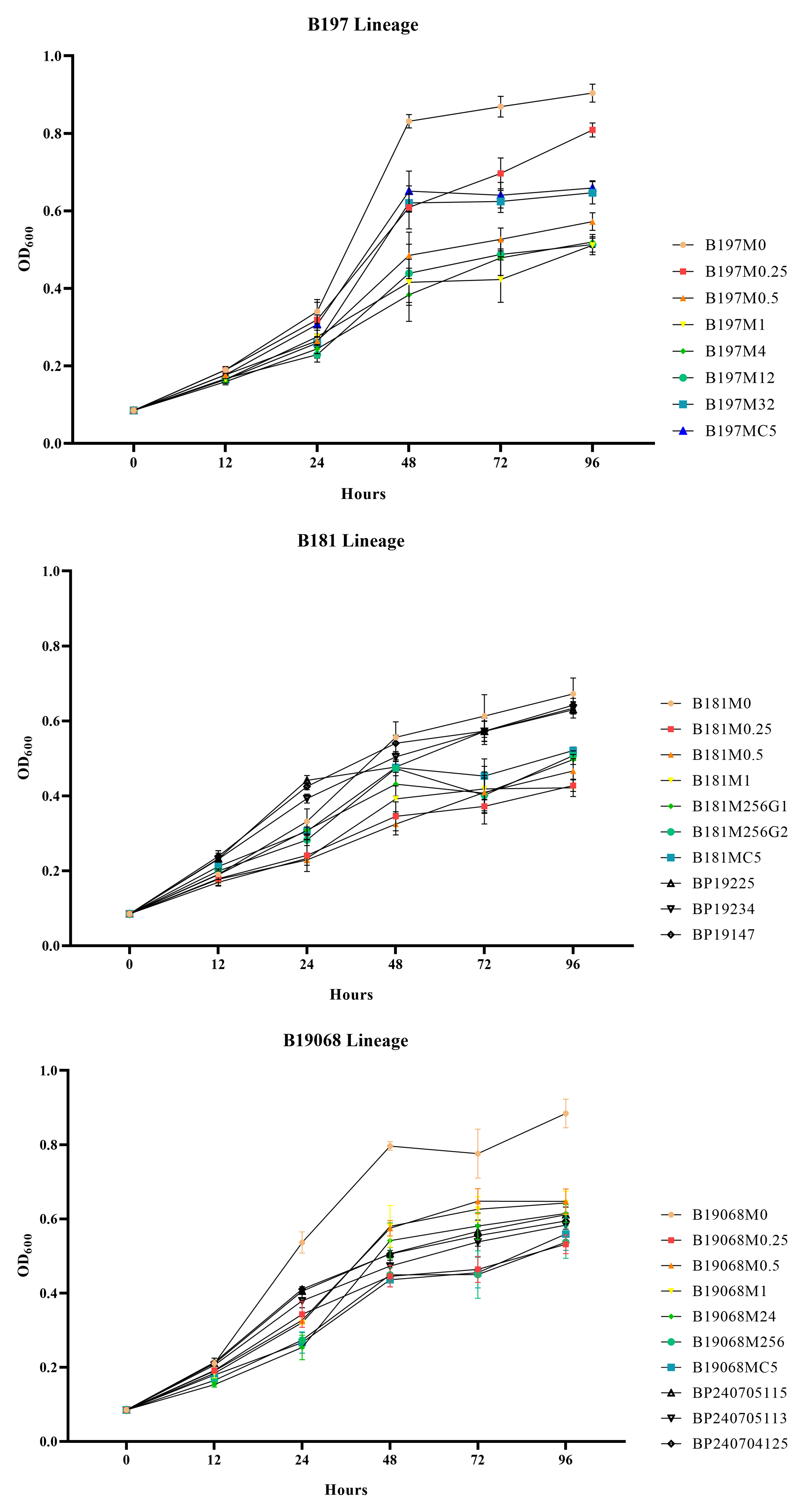

Supplement: SUPPLEMENTARY FIGURE S1 — Raw OD600 growth curves of antibiotic-exposed lineages from the three genetic backgrounds. [file Image_1.tif]
